# Integrating Participatory Social Innovation Into Requirements Engineering for AI Health Care Solutions: Case Study

**DOI:** 10.2196/87984

**Published:** 2026-07-03

**Authors:** Carina Dantas, Miriam Cabrita, Maciej Bobowicz, Harm op den Akker, Xavier Rafael-Palou, Tuukka Hakkarainen, Ira Haavisto, Fredrik Strand, Luis Marti-Bonmati, Eugen Divjak, Gordana Ivanac, Smriti Joshi, Richard Osuala, Stefanie Charalambous, Apostolia Tsirikoglou, Gloria Ribas, Oliver Díaz, Ana Sofia Carvalho, Elísio Costa

**Affiliations:** 1 SHINE 2Europe (Portugal), Rua Câmara Pestana Lote 3 – 1ºD/F, Coimbra, Coimbra, Portugal, +351936498277; 2 School of Medicine and Biomedical Sciences, University of Porto, Porto, Portugal; 3 2nd Division of Radiology, Medical University of Gdansk, Gdansk, Poland; 4 Quantitative Imaging Biomarkers in Medicine, Quibim SL, Valencia, Spain; 5 Research & Innovation, Nordic Healthcare Group, Espoo, Finland; 6 Department of Oncology and Pathology, Karolinska Institutet, Solna, Sweden; 7 Biomedical Imaging Research Group (GIBI230), La Fe Health Research Institute, Valencia, Spain; 8 Department for Diagnostic and Interventional Radiology, University Hospital Dubrava, University of Zagreb School of Medicine, Zagreb, Croatia; 9 Departament de Matemàtiques i Informàtica, Universitat de Barcelona, Barcelona, Spain; 10 R&D department (Health sector), Maggioli S.p.A – Greek branch, Marousi, Greece; 11 RISE-Health, Competences Centre on Active and Healthy Ageing (Porto4Ageing), Faculty of Pharmacy of University of Porto, Porto, Portugal

**Keywords:** requirements engineering, artificial intelligence, social innovation, multistakeholder engagament, ethics

## Abstract

**Background:**

The successful design and implementation of artificial intelligence (AI)–driven solutions in health care requires early and continuous multidisciplinary and multiprofessional collaboration. However, diverse disciplinary educational backgrounds, varying languages, and cultural or geographic differences can lead to misunderstandings. To bridge this gap, a structured approach to AI requirements specification can facilitate a shared terminology and a deep mutual understanding among stakeholders, serving both as a guide for technological development and as a means of defining clear pathways for clinical implementation. While technical requirements are well-established in traditional technology development domains, this structured approach remains relatively underused within clinical and social science contexts. Consequently, valuable insights derived from participatory and stakeholder-driven approaches are often overlooked, limiting the relevance and trustworthiness of AI systems in health care settings.

**Objective:**

This study presents a methodology for requirements gathering, specification, mapping, and verification, specifically engineered for the complex, multistakeholder environment of clinically applied AI. The methodology was implemented within the specific case of an international multidisciplinary project evaluating an AI-based prediction tool for neoadjuvant chemotherapy treatment response for breast cancer and forming a part of the developed AI validation framework.

**Methods:**

The process for AI requirements gathering, specification, and monitoring included 3 iterative rounds of discussion, engaging nearly 150 social, clinical, technical, ethical, and regulatory experts and patients across Europe, South America, North Africa, and Eurasia. It combines established requirements engineering methods (including the MoSCoW [Must have, Should have, Could have, Will not have] framework) with social innovation techniques to ensure inclusivity and contextual relevance.

**Results:**

A key finding is the successful development of a structured framework integrating participatory social innovation with formal requirements engineering in an international AI health care setting, through a traceable multisource workflow including clinical, ethical, and regulatory aspects. It is supplemented with an extensive list of 184 actionable consensus-based requirements, categorized by stakeholder group, providing valuable insights for AI researchers in the oncology field with the potential to be transferable to other digital health domains. The requirements align with the fairness, universality, traceability, usability, robustness, and explainability in the AI (FUTURE-AI) framework, ensuring the tool is trustworthy and comprehensive from a multistakeholder perspective, and ensuring comprehensive consideration of all elements of FUTURE-AI.

**Conclusions:**

The proposed methodology represents a significant advancement for requirements engineering in digital health by extending traditional technical processes to systematically incorporate nontechnical requirements from diverse global stakeholders. This unified approach is essential for ensuring AI solutions are not only technically robust but also clinically relevant, legally compliant, and socially acceptable.

## Introduction

Integrating artificial intelligence (AI) into health care holds significant promise for enhancing patient outcomes and operational effectiveness. However, despite rapid technological advances, many AI-driven tools face challenges in clinical implementation, often due to insufficient alignment with real-world needs and contexts [1,2]. Achieving the successful and sustainable integration of AI in health care requires the early and continuous involvement of diverse stakeholders, defined as individuals or groups who are affected by or have influence over the development and deployment of AI solutions. In the health care context, key stakeholders include health care professionals, technology developers, hospital administrators, policymakers, regulators, and, critically, patients themselves.

Each group of stakeholders engaged in medical AI solutions brings unique perspectives and priorities to ensure that diverse needs are adequately addressed, making the gathering, harmonization, and implementation of requirements a complex but necessary endeavor [3,4]. Participatory approaches, such as co-design workshops or stakeholder interviews, are essential for gathering different needs, expectations, and challenges. However, achieving meaningful engagement involves more than consultation; it requires structured mechanisms to actively listen and respond inclusively to all parties, ensuring that no voice is marginalized in the decision-making process. For example, a significant challenge in gathering requirements for AI solutions in health care is the ontological misalignment caused by discipline-specific jargon and technical language used by the diverse array of stakeholders, which can lead to misinterpretations, incomplete requirements, or ambiguities in system design [4,5]. Without a clear and shared understanding of key concepts, critical details may be lost in translation, which can impact both the development and deployment of AI-driven solutions. Therefore, establishing a common, unified, standardized glossary and structured dialogue sessions is essential to ensure that all stakeholders accurately contribute to and interpret the requirements of the final AI solution [6].

A pressing challenge in the requirement elicitation process lies in the interplay among technical, regulatory, and processual considerations. From a technical perspective, interoperability is a significant hurdle, as ensuring compatibility between legacy infrastructure and cutting-edge solutions can be costly and time-consuming, often requiring tailored integration strategies. Important consideration is given to the technical requirements for building AI tools and ensuring clinical workflow integration and interoperability, specifically how AI systems seamlessly connect with existing electronic health records and other health care infrastructures (eg, picture archiving and communication systems). The dynamic nature of clinical data and deployment environments adds a layer of complexity, as initial assumptions for machine learning models, such as those related to data distributions, imaging protocols, hardware configurations, or data transfer protocols, may quickly become obsolete due to changes in clinical practice, technological upgrades, or shifts in patient population demographics. While models such as the Technology Acceptance Model [7] and the Unified Theory of Acceptance and Use of Technology [8] are widely used to explore anticipated user perceptions in digital health, they offer limited support for translating stakeholder input into actionable requirements during early-stage design. Regulatory and procedural dimensions further complicate the landscape, and harmonizing international and national regulations requires a nuanced understanding of legal frameworks, especially in sectors subject to rigid compliance requirements, such as health care. Ethical considerations and bias mitigation are also crucial, as AI systems must be designed to prevent inequitable health care outcomes by addressing different biases, either individual, technical, organizational, or societal [9]. Finally, although technical guidelines such as fairness, universality, traceability, usability, robustness, and explainability in AI (FUTURE-AI) [10] have recently appeared to support medical trustworthy AI technology, user acceptance and change management play a significant role, as health care policymakers and professionals may resist AI adoption due to multiple concerns [11,12].

Addressing these challenges demands a structured and iterative approach to requirements engineering, incorporating continuous stakeholder engagement, robust data governance, and adaptive methodologies to accommodate the evolving nature of AI technologies in health care.

Requirements engineering provides the methods to systematically specify and validate requirements gathered from different sources, including stakeholder consultation [13]. However, while widely used in software development, requirements engineering techniques for specifying and validating requirements remain underused in the clinical context [14], where they could play a critical role in enhancing the trustworthiness, usability, and adoption of AI systems. The study presented in this article fills that need by presenting a method and use case for requirements elicitation, specification, and validation, integrating participatory social innovation (SI) with formal requirements engineering. Besides the novelty of the method, the work presented proposes a traceable multisource workflow, including clinical, ethical, and regulatory aspects, which was validated and is fit to be used in international AI–health care settings.

Drawing on the work performed within the International Clinical Validation of Radiomics Artificial Intelligence for Breast Cancer Treatment Planning (RadioVal) project [15], which aims to validate AI predictive tools for breast cancer treatment, we present a multidisciplinary framework for the systematic elicitation, management, and continuous refinement of stakeholder requirements in the development of predictive AI technologies, with a focus on oncology. The framework presented in this article is intended to support the development of clinically robust, ethically sound, and legally compliant AI systems by integrating clinical, socioethical, regulatory, and end user perspectives from the outset.

The proposed approach emphasizes early stakeholder engagement to ensure alignment with principles of safety, fairness, accountability, and clinical utility. Its primary objective is to demonstrate how a participatory, multistakeholder process can strengthen the relevance and acceptability of AI system requirements. We outline a replicable methodology that combines desk research, contextual and document analysis, structured workshops, and formal elicitation techniques, offering practical guidance for similar digital health initiatives. The process encompasses the full workflow of requirements engineering, including predevelopment elicitation, predeployment validation, postdeployment monitoring, and adaptive requirement revision over time.

A secondary objective of this work is to provide an open access, comprehensive catalog of stakeholder requirements, based on a large-scale, multicountry study. This resource is intended to inform future research and development efforts by enabling rapid contextual adaptation across diverse health care settings. Ultimately, this work aims to promote the broader adoption of responsible AI principles in health care, supporting the development of AI tools that are not only technically effective but also socially and clinically acceptable and aligned with patient-centered care.

## Methods

### Overview

This study describes structured research and processes carried out within the European Union (EU)–funded RadioVal Project between 2022 and 2024. RadioVal is an EU-funded Research and Innovation Action (2022‐2026) developing an actionable framework for the international validation of radiomics-based tools in treatment response prediction for neoadjuvant breast cancer, with scalability potential to other use cases [15]. It is also implementing an international clinical validation study of radiomics-based prediction of neoadjuvant treatment response from breast magnetic resonance imaging. RadioVal introduces new tools to enable transparent and continuous evaluation and monitoring of radiomics tools over time based on FUTURE-AI principles, fairness, universality, traceability, usability, reproducibility, and explainability [10]. The consortium is constituted by 16 international research and development institutions, academic organizations, health care organizations, experts in SI and participatory democracy, experts on value-based care, cost-effectiveness analysis, and regulatory aspects, as well as small and medium-sized enterprises from 13 countries covering a broad range of expertise in health care, AI development, validation, deployment, and monitoring of AI software. The project is based on an international clinical network, covering Southern, Northern, Central-Western, and Eastern Europe, as well as South America, Eurasia, and North Africa. The extensive expertise of the project partners enabled a comprehensive validation of AI-driven tools for predicting treatment response to neoadjuvant chemotherapy in breast cancer. Accordingly, by specifying the needs of the multinational and multidisciplinary stakeholders and ensuring compliance with contractual obligations as well as international regulatory frameworks, we developed a methodology for the systematic gathering, specification, and validation of requirements.

The methodological process presented in this paper considered the 21-item list of the Standards for Reporting Qualitative Research [16], with the aim of supporting transparency and rigor in the multistakeholder and participatory components of requirement elicitation.

To ensure conceptual clarity, below we provide the definitions of key terms used in this article. The requirements classification schema is based on the BABOK Guide, a widely recognized international standard for business analysis, encompassing business, stakeholder, and solution requirements [17]. In addition, regulatory requirements were incorporated to address the objectives of this work. The definitions presented herein are partly derived from existing literature and, where no established definition was identified, developed by the authors.

- Need: a wish or desire from a certain stakeholder either in relation to the future solution being developed or related to the stakeholder’s current situation. Needs are often not clearly defined and may be ambiguous, yet form a clear basis for defining stakeholder requirements.
- Requirements: refer to detailed and structured descriptions of what a technology system or tool must achieve or include to be considered complete and functional. These are typically gathered at the initial stages of development and serve as the foundation for design, implementation, testing, and evaluation.
- Stakeholders are a group or individual with a relationship to the change, the need, or the solution being realized. They are often defined in terms of interest in, impact on, and influence over the change. In the context of the RadioVal project, we identified 7 major groups through a comprehensive stakeholder mapping, including patients and carers, health care professionals, hospital administration, ethicists and regulators, policymakers, AI developers, and payors [18].
- Stakeholder requirement: International Organization for Standardization (ISO)/International Electrotechnical Commission (IEC)/Institute of Electrical and Electronics Engineers (IEEE) 15288 standard (chapter 6.4.2) [19] defines a stakeholder requirement as “a need or expectation that a stakeholder group (eg, user, operator, or regulator) has for a system, and the way it needs to be able to interact with its operational environment to meet those needs.” These requirements serve as the basis for validating the system’s operational capabilities.
- Solution requirements: statements that describe the characteristics a particular solution must have to meet the needs of stakeholders and business. They are often subdivided into functional and nonfunctional requirements, or into further subdivisions. In contrast to business requirements (which can be very high-level) and stakeholder requirements (which can describe wishes, needs, or emotions), solution requirements are designed to be more concrete, unambiguous, and verifiable statements that directly lead to the technical designs and implementations of the solution.
- Business requirements: high-level statements that describe the business’s goals and objectives at the enterprise level. In a European R&I project, a diverse group of organizations temporarily come together to achieve a common set of goals. The goals are laid out in a contract (eg, the Grant Agreement [GA]) between the consortium members and the funding agency (the European Commission). The GA is a legal document that specifies the terms under which research funding is provided by the funding agency, including the scope of work, deliverables, reporting requirements, and compliance expectations, without the expectancy of commercial return. It corresponds to a master service agreement or contractual agreement, such as a partnership agreement or a funding agreement in business. Thus, the grant agreement was the starting point for the requirements collection process.
- Regulatory requirements: either high-level or more detailed stipulations derived from official regulatory documents. They describe, or more frequently refer to, applicable regulations.

**Figure 1.**
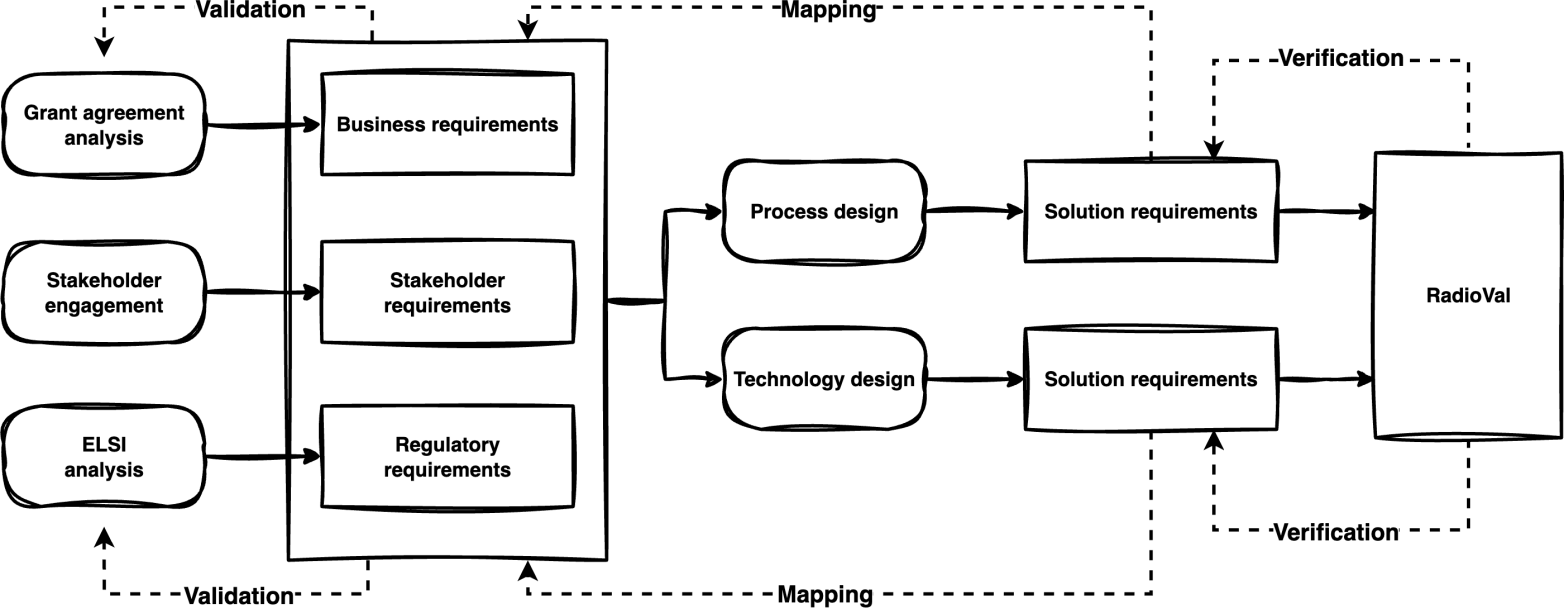
The requirements elicitation, specification, validation, mapping, and verification process in RadioVal. ELSI: ethical, legal, and social implications.

The participatory activities and requirements engineering processes were explicitly framed around FUTURE-AI principles. Workshops, interviews, and deliberative sessions were structured to elicit stakeholder perspectives aligned with these dimensions. For example, this included identifying user requirements related to usability, discussing transparency and communication needs linked to explainability, and addressing ethical and societal considerations associated with fairness and general governance principles. Figure 1 shows a schematic of the overall process that was followed for eliciting the requirements, deriving solution requirements, and performing validation and verification. In this context, validation refers to confirming that stakeholder requirements accurately reflect user needs (“Are we building the right thing?”), whereas verification assesses whether the implemented solution meets the specified requirements (“Are we building it right?”).

### Requirements (Predevelopment) Elicitation

When analyzing the flow depicted in Figure 1, 3 primary sources of requirements gathering can be identified: the GA, stakeholder engagement activities, and ethical, legal, and social implications (ELSI) investigation.

A detailed analysis of the project’s GA served as the basis for defining business requirements. This process was carried out by a project manager with extensive expertise in requirements engineering as well as experience with European funding and the structure of GA documents.

A combination of stakeholder engagement activities, including surveys, workshops, and interviews, was used to derive stakeholder requirements (Table 1). When necessary, literature reviews were conducted in preparation for the participatory activities to provide guidance and framing. First, a comprehensive survey on trustworthy AI in breast cancer was performed among clinicians in collaboration between the technical and clinical teams of the project, as described in [20]. Two focus groups were conducted with patients with breast cancer survivor associations to integrate patients’ perspectives. In parallel, multidisciplinary viewpoints were gathered through 3 rounds of multistakeholder workshops, each adapted to the evolving maturity of the solution. The first round used an exploratory science café format, the second centered on ideation, and the third and final round focused on prototype deliberation. The methods and results of these activities are detailed in [18]. The findings were complemented by 6 in-depth interviews with experts in the areas of policy making in health care, insurance, and ethics as well as a patient representative.

**Table 1.** Stakeholder activities performed in the RadioVal project for requirements gathering, including the number of stakeholder representatives per activity.

Type of activity	Participants (n=148), (n)
Comprehensive survey on trustworthy AI^a^ in breast cancer	23 (health care professionals)
Focus groups with patients	16 (patients and carers)
Multistakeholder workshops	103 (patients and carers n=23; health care professionals n=30; hospital administration n=7; ethicists and regulators n=15; policymakers n=2; AI developers n=26)
Semistructured interviews on socioethical issues	6 (patients and carers n=1; ethicists and regulators n=2; policymakers n=2; payors n=1)

a AI: artificial intelligence.

### Ethical Considerations

The University of Porto’s Ethics Committee approved the study under reference number (2024/CE/P19P422/2023/CETI). Informed consent procedures were adequately applied throughout the study, which has also adhered to local, national, regional, and international law and regulations regarding protection of personal information, privacy, and human rights. No type of compensation was provided to the participants in the study. Besides formal ethics requirements, stakeholder participation introduces additional ethical considerations that merit further reflection. Power dynamics among participating actors, particularly differences in institutional authority, technical expertise, and access to resources, may have shaped deliberation processes and influenced how requirements were articulated and prioritized. Despite facilitation efforts aimed at fostering inclusive participation, some stakeholder voices may have carried greater implicit weight during collective decision-making. Table 1 summarizes stakeholder participation in the engagement activities.

Additionally, a detailed review was conducted of 3 international clinical guidelines in breast neo-adjuvant chemotherapy, most frequently used by the clinical partners in the consortium—those of the European Society of Medical Oncology, the American Society of Clinical Oncology, and the American College of Radiology—to inform the engagement of health care professionals. Finally, a scoping review was performed, focusing on scientific and gray literature on potential sources of bias and unequal treatment of patients when using radiomics models in breast cancer.

Finally, several activities of contextual and documentary analysis were undertaken to support the ethical, legal, and social implications examination, ultimately informing the regulatory requirements. A general overview of legal, regulatory, and policy frameworks relevant to AI-driven tools in health care, both within and outside the EU, was developed. Particular attention was given to liability, addressed through desk research and the examination of 4 key EU regulations in this context, including the AI Liability Directive, the Product Liability Directive, the AI Act, and the Medical Device Regulation (MDR). This analysis, performed by the project’s legal experts, concluded with a set of reflection points for further discussion and consideration.

### Requirements Specification

All sources described above during the requirements elicitation step were systematically analyzed by a multidisciplinary team with expertise in requirements engineering and digital health. Insights derived from this process were formalized as requirements using a structured template adapted from Van Velsen et al [3], which includes the following fields: ID, type, requirement description, rationale, source, priority, author, status (proposed, clarified, accepted, or rejected), and revision history.

The process started with an analysis of the GA to derive high-level business requirements, such as “GA.1: RadioVal shall validate the real-world applicability of predicting neoadjuvant chemotherapy outcomes in women with breast cancer using artificial intelligence approaches based on radiomics,” referencing the specific section in the GA document on which the requirement was based. Requirements that provide additional details are codified in a sequential way, such as “GA.1.2: The RadioVal tool shall be tested with at least 2700 cases with neoadjuvant chemotherapy, magnetic resonance imaging, and follow-up information,” which provides further details to the project’s “core” objective in GA 1. The whole process of extracting business requirements from the GA document should be straightforward, as the GA is meant to codify the objectives of the research project in verifiable ways. Any cases of unclear or contradictory statements in the GA—which are unfortunately not uncommon—are typically clarified in project-level discussions at the project’s onset.

The systematic analysis of qualitative results emerging from the various stakeholder activities listed in Table 1 was performed by 2 researchers (MC and HA), an expert in requirements engineering who drafted the stakeholder requirements based on the materials and the second researcher responsible for drafting the initial minutes of the activities to assist in clarifying any doubts.

The qualitative materials were systematically transformed into formal requirements by applying the FUTURE-AI guidelines as the a priori coding framework. The data were segmented into meaningful units and deductively coded against the core FUTURE-AI principles, such as fairness, universality, traceability, usability, robustness, explainability, safety, and accountability. These coded segments were then analyzed to identify recurring patterns and thematic constructs within each principle, thereby ensuring that stakeholder concerns were explicitly aligned with recognized ethical and technical domains. Subsequently, each theme was translated into structured requirements by articulating the implied system behaviors, constraints, or quality attributes in a formalized manner, for example, by specifying functional capabilities or nonfunctional criteria such as performance, transparency, or safety thresholds.

To ensure a correct interpretation of qualitative data and a correct translation into requirements, codifying the source, rationale, and author fields of each requirement allows issues identified during the next steps of refining the requirements. For example, “Author A, requirement SI.10 states B, based on statements from health care professionals in document C, but I believe during that discussion they meant to say D.” The process allows for such discussions to take place at any time during the requirements, design, or development process, as requirements are always fluid.

The initial set of requirements derived in this way was iteratively refined through feedback from the multistakeholder engagement lead, the clinical lead, and the technical lead, who also resolved any disagreements between the 2 researchers (MC and HA) leading the requirements analysis. For prioritization, the MoSCoW (Must have, Should have, Could have, Will not have) method [21] was applied to the specific context of the project, which facilitated transparent decision-making. Prioritization was provided by 5 different experts from the clinical, stakeholder engagement, and technical domains in the project, and an average score was calculated by assigning the MoSCoW classifications with scores of +2, +1, 0, and –1. Then, averaged values of 1.5 or higher were considered “Must Have,” values between 0.5 and 1.4 are “Should Have,” etc. These prioritized inputs were then systematically translated into solution requirements through process design and technology design.

### Requirements (Predeployment) Validation

Three main processes ensure the correctness of the requirements specified. From left to right in Figure 1, the first step, validation, is the process of confirming that the original requirements capture the needs of the stakeholders. As business requirements and regulatory requirements should generally not be subject to interpretation, this process focuses on stakeholder requirements and aims to answer the question: “Do our Stakeholder requirements accurately capture what the stakeholders want?” Requirements validation is generally performed in 2 complementary phases. First, once the initial set of stakeholder requirements are defined by the requirements engineer, they are immediately validated with representatives involved in the stakeholder engagement process. This ensures that the source material has been correctly interpreted. Second, validation is treated as a continuous activity, similar to subsequent mapping and verification steps. Because stakeholder engagement extends beyond the elicitation phase, new insights may challenge the validity of previously specified requirements. It is therefore essential to conduct iterative validation at key stages of the development process (eg, during prototype testing with end users).

### Requirements Mapping

By mapping all solution requirements to one or more business, stakeholder, or regulatory requirements, it is possible to ensure that each of those latter requirements has indeed led to a specific element of the solution that addresses the requirement. It answers the question, ”Were all our original requirements considered when defining solution requirements?” In requirements engineering, projects aimed at developing a single market-ready tool with a clearly defined scope typically allow for a direct mapping of solution requirements onto business and stakeholder requirements, leaving few gaps. However, the scope of the RadioVal project extends well beyond the delivery of a single tool, encompassing a diverse range of outputs, including AI-driven tools, knowledge resources, and policy recommendations. Given this multifaceted nature, it was neither feasible nor expected that every business, stakeholder, or regulatory requirement would correspond directly to a clearly articulated set of solution requirements. When appropriate, requirements were systematically mapped to corresponding technology modules and activities, with responsibilities assigned to designated module owners. The mapping was guided by a detailed activity map depicting the project’s modular structure, task sequences, interdependencies, and partner roles. Modules and activities were further broken down into components and subcomponents. To ensure consistency, traceability, and verifiability, all contributors followed a standardized structure for solution requirements, categorizing them as functional, nonfunctional, or integration-related. A collaborative session with practical examples facilitated shared understanding and alignment across teams.

### Requirements Verification

From left to right in Figure 1, verification is the final process of confirming that the developed solution matches its defined specifications (ie, the solution requirements). It answers the question, “Did we build what we set out to build?” The methods used to verify the correct implementation of solution requirements vary considerably depending on the nature of the requirement. For example, technical nonfunctional requirements, such as “User account information must be stored encrypted in a MySQL database,” can be verified directly by technical inspection. More complex performance-related requirements, for instance, “The system must be able to handle up to 100 login attempts per minute and provide an access token within 500 ms for each correct request,” necessitate dedicated test software to simulate user activity and measure system response. By contrast, some user interface requirements can be verified through simple observation. For example, “On every page of the web portal, a logout button should be present at a consistent location” can be verified by manually navigating through the application and confirming its presence. This tiered approach to verification ensures that solution requirements are validated comprehensively across technical, functional, and usability dimensions.

### Requirements Monitoring

All 3 of these processes defined above should be appropriately managed within any project in a requirements monitoring process. To ensure that all specified requirements are fulfilled according to their assigned priority, the requirements implementation monitoring followed a combination of 2 approaches, which include the postdeployment monitoring and the adaptive requirement revision over time.

#### Formal Tracking of Requirements

As described above and illustrated in Figure 1, the preferred method for tracking requirements follow-up is to generate solution requirements and map these onto the business, regulatory, and stakeholder requirements. As the implementation of the solution requirements is being followed up, progress can be tracked against the original requirements. For all business, regulatory, and stakeholder requirements which are not mapped in this way, the next method was used.

#### Qualitative Periodic Review

For requirements where no precise solution requirements are directly linked, a periodic, qualitative review of their progress was performed. This process identified (1) the progress on the implementation in a narrative, descriptive way, (2) the lead responsible partner and/or individual, and (3) a categorical status indicator as one of: unknown, in progress, blocked, or complete. A tracking sheet was created with all relevant information related to the requirement (eg, source, rationale, priority, author’s initials, status, and the history log), as well as the responsible person and status.

Based on this methodology, the process used for defining, gathering consensus, and prioritizing requirements in RadioVal is presented in the following section.

## Results

### Requirements Elicitation, Specification, and Validation

A total of 218 requirements were initially specified, after which an iterative consensus-building process was conducted to refine their clarity, feasibility, and relevance. In line with best practices in requirements engineering, multiple rounds of discussions were held with the project’s requirements engineers as well as technical and clinical leads. During this process, requirements were rephrased, clarified, or, where appropriate, rejected, resulting in a final set of 184 requirements (Table 2). In this process, any similar or duplicate requirements were clustered and merged by identifying shared intent and then rewritten as single, unambiguous requirements with clear scope, in line with the guidance provided in widely accepted standards, such as the ISO/IEC/IEEE 29148:2018.

**Table 2.** Number of requirements specified and accepted by type, along with their distribution per priority category.

Type of requirement	Specified (n=218)	Accepted (n=184)	Priority
Business requirements	44	44	Must have: 38 Should have: 6
Stakeholder requirements	158	125	Must have: 40 Should have: 37 Could have: 33 Will not have: 15
Regulatory requirements	16	15	Must have: 7 Should have: 8

Rejected requirements were determined to be either outside the defined scope of the project or infeasible to implement within existing technical and financial constraints. Requirements deemed out of scope were those that did not align with the project objectives or intended system boundaries, while those considered infeasible reflected limitations in available technology, resource capacity, or budgetary provision. This assessment ensured that the final set of requirements remained both relevant to the project aims and achievable within the practical constraints of development and deployment.

To provide a transparent audit trail from original inputs to consolidated requirements, all reviews were documented. The complete list of requirements is provided in Multimedia Appendix 1.

First, from the analysis of the RadioVal project’s GA, 44 business requirements were identified (eg, GA.2: “RadioVal shall deliver a methodological framework to evaluate radiomics AI based on four stages: feasibility, capability, usability, and applicability”), of which 38 were prioritized as “Must Have” and 6 as “Should Have.” Then, the stakeholder engagement activities and desk research led to 158 stakeholder requirements (eg, SI.27: “Patients want that the RadioVal study be approved by an ethical and legal committee before commencing, ensuring safety, privacy, and confidence of patients”), of which 125 were accepted. Among these, 40 were prioritized as “Must have,” 37 as “Should have,” and 33 as “Could have.” Finally, 16 regulatory requirements were elicited (eg, MDR.1.1: “In order to comply with MDR, a quality management system should be established according to ISO 13485 and IEC 62304.”), of which 15 were accepted, with 7 categorized as “Must Have” and 8 as “Should Have.”

### Requirements Mapping, Verification, and Monitoring

**Figure 2.**
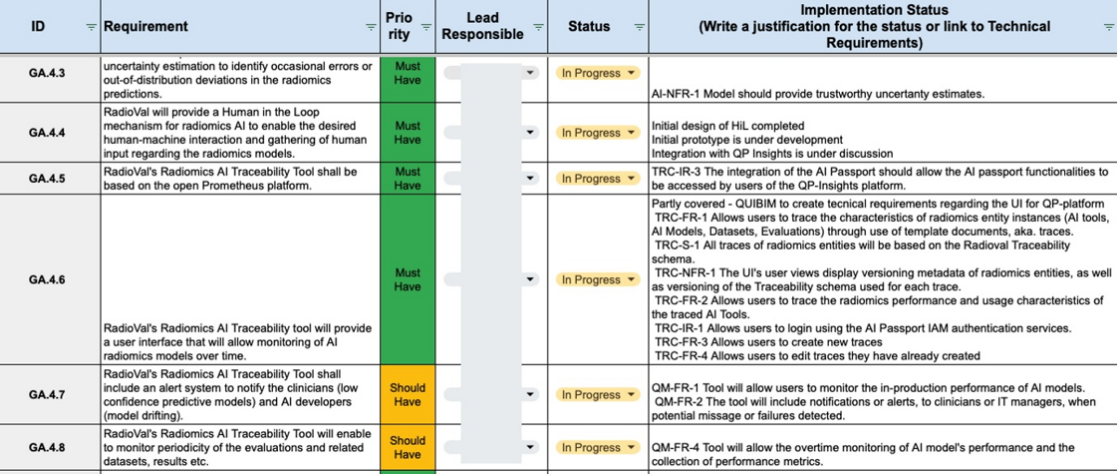
Screenshot of the qualitative periodic requirements monitoring tracker.

A total of 80 solution requirements were defined in alignment with the overarching business, stakeholder, and regulatory needs. Each requirement was systematically documented to ensure consistency, traceability, and effective management throughout the development lifecycle. The documentation process used a standardized structure comprising a unique identifier, associated component and subcomponent (where applicable), a detailed requirement description, the requirement type, and a priority level determined according to the MoSCoW prioritization method. The requirements were distributed across several categories, namely functional, nonfunctional, integration, and documentation and specification requirements. This structured approach facilitated precise alignment between technical specifications and solution components, promoting transparency in requirement management and ensuring that the developed solution effectively addressed all critical technical and operational objectives. A qualitative, periodic requirements monitoring tracker was established to systematically oversee the implementation of the requirements classified as “Must Have” or “Should Have” (Figure 2). The tracker is structured to capture essential information for each requirement, including its unique identifier, description, priority level, the partner responsible for implementation, current status (at risk, in progress, and fulfilled), and a justification for the status assignment. By periodically reviewing and updating this tracker, the project team can identify potential risks early, monitor ongoing progress, and ensure timely intervention when necessary. This approach facilitates the proactive management of technical and operational challenges, supports accountability among consortium partners, and enables project leadership to maintain alignment between the evolving development of tools and the initially defined requirements. Importantly, the tracker is maintained as a living document throughout the entire project lifecycle, allowing it to reflect changes in priorities, emerging stakeholder insights, and iterative refinements in the solution design.

In terms of the discussion and validation process, it is worth mentioning that 16 requirements were significantly rephrased or further developed by the researchers’ team, due to disagreements and/or discussion, while 116 requirements were significantly revised in content or prioritization following stakeholder workshops, and 3 were specifically derived from interviews with regulatory experts. The source and history of revisions of each requirement are documented in the Multimedia Appendix 1.

**Figure 3.**
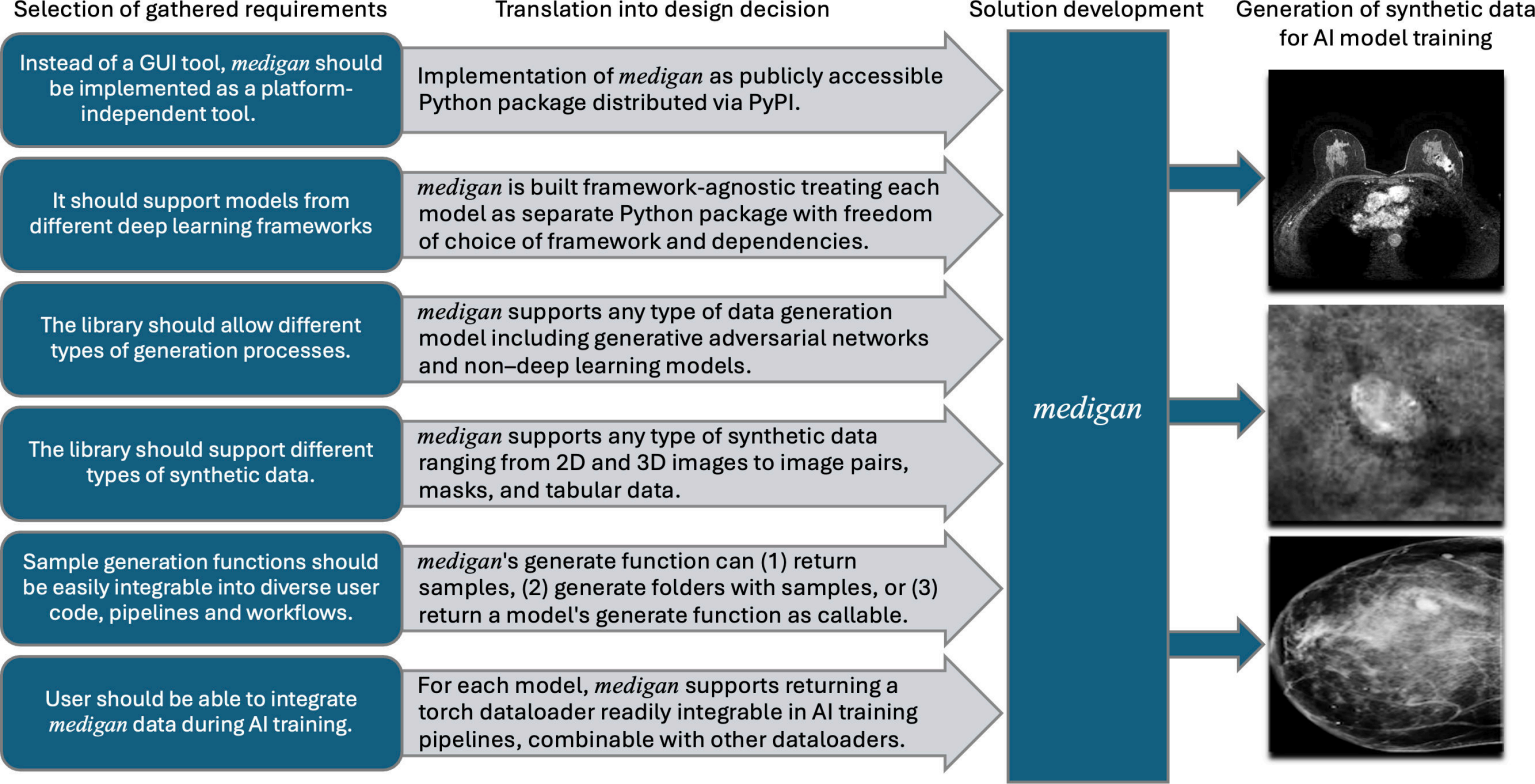
The requirements gathering and translation into technical design decisions for the development of the *medigan* library within the RadioVal project. AI: artificial intelligence; GUI: graphical user interface.

A practical example of how requirements were mapped to implemented technical design decisions in the RadioVal project is the *medigan* library [22]. The development of *medigan* followed the technical requirements methodology from the RadioVal project, translating stakeholder requirements into specific design decisions. As of October 2025, the *medigan* library has achieved tangible community, educational, and scholarly impact, with approximately 185 GitHub (GitHub) stars, 21 forks, and around 45 academic citations. In the requirements gathering process, the discussions highlighted a preference for a Python (Python Software Foundation)-based, importable library and the need for flexibility in supporting a variety of medical image generative models, particularly in the context of the RadioVal breast imaging use cases. In response, *medigan* was designed to be framework-agnostic, ensuring seamless integration into AI training pipelines. The requirements were categorized into functional, nonfunctional, and integration-related needs, with priorities set to address the most critical aspects first. This structured approach resulted in clear, traceable specifications that guided the development of *medigan*, ensuring ease of contribution, search, and execution of generative models. Furthermore, as part of the structured requirements gathering process, the *medigan* library was designed to align with the FUTURE-AI guidelines [10], particularly the principle of traceability, emphasizing model transparency and reproducibility. To support this, *medigan* integrates respective documentation and clear version-controlled metadata for each of its generative models, ensuring traceability and thereby fostering trust and accountability in the resulting synthetic medical data. Overall, the iterative, user-driven process facilitated continuous refinement of the library’s design to meet user expectations and ensure technical feasibility. The final framework for *medigan* aligns user needs with technical capabilities, as detailed in Figure 3, which summarizes key user requirements and their corresponding design decisions for synthetic data generation to support AI model training.

## Discussion

This study presents a methodology for systematically specifying and monitoring requirements in complex digital health projects, exemplified by the international RadioVal project, which aims to validate AI-driven tools for predicting breast cancer treatment response. Over 200 requirements were initially specified and iteratively refined through discussions among requirements engineers, technical partners, and clinical experts, resulting in a final set of 184 requirements: 44 business (reflecting the overall goals of the project, eg, “The RadioVal tool’s performance shall be calibrated with at least 4450 cases”), 125 stakeholder (on the needs of the different stakeholder groups, eg, “A patient wants to understand, at all times, which treatment options are available to them”), and 15 regulatory requirements (defining needs and constraints from legislation, eg, “In order to comply with MDR, a quality management system should be established according to ISO 13485 and IEC 62304”). This set represents a snapshot as of August 2024, acknowledging that requirements and prioritization may evolve with emerging insights.

Prioritization of requirements posed several important challenges. Business requirements, derived directly from the GA, reflect the core objectives of the project and are generally prioritized highly. However, stakeholder input may occasionally justify deviations from the original business objectives, or, otherwise, final prioritization might include limited attention to some stakeholder input if they fall outside of the project’s defined scope and timeline. For example, requirement PINK.26 derived from focus groups with “Pink Ribbon”, the young cancer patient organisation in Poland, related to providing patients with information on further management or potential clinical trial participation, was classified as “Will not Have.” While clearly a valuable concept, implementing it would demand substantial resources not included in the project scope, illustrating the trade-offs inherent to expectations management. Importantly, even stakeholder requirements not prioritized as “Must Have” or “Should Have” provide rich insight that can inform the development of medical AI tools in broader contexts. Regulatory requirements, in turn, reflect evolving standards for AI in health care. While immediate MDR approval was not expected, prioritization focused on preparatory actions such as technical documentation and quality control systems to support eventual compliance.

Consensus during the MoSCoW prioritization process was generally high, with 76 out of 184 (41%) of requirements being prioritized without disagreement. Disagreements primarily reflected 3 categories of tension: clinical feasibility, technical complexity, and perceived patient value. For the remaining requirements, disagreement could be considered minimal. For each requirement that was prioritized by 5 different experts, we define the contentiousness score as the minimum number of steps it would take to align the views of all experts (one step being eg, a change from “Must Have” to “Should Have,” while a change from “Must Have” to “Could Have” would represent 2 steps). Table 3 shows the occurrences of the different contentiousness scores.

**Table 3.** Contentiousness scores.

Contentiousness score	0	1	2	3	4
Occurrence	76	50	48	9	1

The requirement with the highest contention among the 5 experts was, “SI.33: Prediction bias of the RadioVal algorithm should be prevented between individual patients of different races.” In this case, 3 experts prioritized the requirement as “Must Have” (on the principal basis that bias toward race should be prevented), whereas 2 experts prioritized the requirement only as “Could Have” (since fulfilling this requirement would be difficult due to the lack of racial data in the available training sets).

Another example of a contentious requirement was, “MDR.2: in order to acquire CE (Conformité Européenne) certification, the Clinical Evaluations and Clinical Investigations should be performed according to MDR” (a legal requirement), with a contentiousness score of 3; it shows a similar difference in prioritization related to some prioritizing based on importance, while others take a more realistic viewpoint (CE certification is almost certainly out of scope of the project).

Translating business, stakeholder, and regulatory requirements into actionable solution requirements is an ongoing, iterative process throughout the development timeline. This approach strikes a balance between research-driven innovation and practical feasibility, ensuring alignment between technical implementation, clinical utility, regulatory compliance, and stakeholder needs. For example, “requirement SI.17: healthcare professionals want to receive training (inc material, time) on how to use RadioVal in their workflow,” can be linked to measurable usability outcomes, as adequate training is expected to reduce cognitive workload, improve task efficiency, and increase user satisfaction when interacting with the system.

The deployment of AI in health care implies access to vast amounts of sensitive patient data, raising significant privacy and security issues and ensuring compliance with regulations, such as the General Data Protection Regulation, which adds layers of complexity to technical requirements. The importance of robust governance frameworks to navigate the ethical and regulatory challenges associated with AI implementation in clinical settings has been widely emphasized [23]. Additionally, conventional and static approaches in requirements engineering for AI systems are ill-suited to the evolving characteristics of health care data and clinical settings. To ensure sustained performance and clinical relevance, it is essential to adopt continuous monitoring and iterative refinement of the system requirements [24]. Failure to address temporal drifts or emergent biases in the data can lead to compromised model performance and inequitable health care outcomes. Mitigating these biases requires a meticulous and ongoing process of requirements specification that prioritizes fairness, accountability, and ethical considerations toward a more transparent and trustworthy AI system.

The introduction of a structured process for eliciting requirements in the development of AI solutions for health care adds substantial value to the scientific body of knowledge by systematizing an often fragmented and opaque stage of innovation. Current evidence suggests that poorly defined or inadequately validated requirements often result in AI tools that lack clinical utility, fail to integrate with existing workflows, or do not meet regulatory and ethical standards. By establishing a rigorous, reproducible methodology, underpinned by stakeholder participation across clinical, technical, regulatory, and patient communities, researchers can ensure that requirements are not only technically sound but also socially and contextually relevant. This participatory approach fosters trust and transparency, enhances the interpretability of design choices, and generates empirical insights that contribute to the broader discourse on responsible and human-centered AI in health.

Beyond its immediate application, the process demonstrates high transferability to other AI-enabled health domains and, more generally, to data-driven solutions in regulated environments. Its structured nature allows adaptation across diverse clinical settings and technological contexts, supporting scalability while maintaining methodological integrity. The explicit inclusion of stakeholders throughout the elicitation process ensures that domain-specific nuances, ethical priorities, and implementation barriers are consistently captured, and such principles are equally critical in other sectors, such as public health, social care, and digital therapeutics. Finally, in contrast to acceptance models such as the Technology Acceptance Model or Unified Theory of Acceptance and Use of Technology, which identify determinants of anticipated or actual use, our proposed approach supports the systematic translation of multistakeholder input into structured and prioritized system requirements. In this sense, it complements these models while extending them toward actionable design decisions in complex health care settings, including organizational and ethical dimensions often underrepresented in perception-based frameworks. Consequently, this framework not only strengthens the scientific foundations of requirement engineering in health care AI but also offers a transferable blueprint for participatory design and governance that may support the development of trustworthy AI systems across disciplines.

While several European initiatives have developed methodologies to ensure that AI solutions are safe, effective, and aligned with clinical needs, published evidence from large-scale, multicountry projects remains limited, with many initiatives still in progress and requirements yet to be reported. This manuscript addresses that gap by describing the applied methodology and providing a comprehensive, reproducible list of requirements.

It is important to acknowledge that the recruitment strategy applied for the stakeholder consultation and interviews with experts was based on a convenience sampling, which may generate biases in the results collected and reflects a limitation of the study. This approach resulted in some imbalance in stakeholder representation, with comparatively limited input from policymakers and payors and with patient perspectives incorporated indirectly rather than through direct participation in the prioritization process. While these factors may have influenced the resulting requirement set and its prioritization, for example, by placing greater emphasis on the perspectives most strongly represented, the approach was considered appropriate given the exploratory nature of the work and the practical constraints associated with accessing a broad range of stakeholders. Efforts were made to include diverse viewpoints within these constraints, and the findings still provide a valuable and grounded foundation for requirement development. Nevertheless, the results should be interpreted with an awareness of these limitations, and future work could build on this by engaging a more balanced and comprehensive stakeholder sample to further validate and refine the outcomes.

Moreover, the interpretation, specification, and prioritization of requirements are inherently subjective processes and potential sources of conflict. During group discussions involving patients, health care professionals, and other experts, the actual needs of stakeholders are not always explicit and often require interpretation, which can introduce errors in requirement definition. Conflicts can also emerge both across and within stakeholder groups. For example, in 2 focus groups with patient associations representing different age groups (young adults vs older adults), it became evident that the needs, concerns, and challenges of patients with breast cancer and survivors vary considerably depending on age and other contextual factors.

Furthermore, prioritization itself can be contentious, as experts may assign different levels of importance to specific requirements. To mitigate this, a democratic procedure was adopted in which individual prioritization scores were converted into numerical values (eg, Must Have=2 or Should Have=1) and averaged across experts to determine the overall priority. This approach balanced divergent perspectives and ensured a transparent and systematic prioritization process. It is acknowledged that the prioritization phase relied primarily on expert consensus using the MoSCoW method, which may introduce cognitive and group decision-making biases. Direct patient participation was limited because requirements were formulated in technical terms that could hinder meaningful engagement at this stage. To mitigate this, a domain expert involved in earlier participatory activities contributed to prioritization to help preserve patient perspectives. Nevertheless, this approach cannot fully replace direct patient involvement and may have reduced the visibility of needs from disadvantaged groups. Additionally, the full list of final requirements was not presented and discussed with all stakeholder groups due to its length and domain expertise, but the most relevant and less consensual formulations were presented at the third SI session and consensualized with the involved stakeholders.

Moreover, culturally driven differences across regional contexts in Europe, South America, and North Africa may have affected communication styles, perceptions of consensus, and expectations regarding participation and authority. These factors could influence both the expression of needs and the willingness of certain stakeholders to challenge dominant perspectives, and although this was considered in the different activities, recognizing these limitations is essential when interpreting the outcomes of multistakeholder requirement elicitation processes. Future applications of the methodology could benefit from more explicit reflexive practices, such as structured facilitation protocols or culturally sensitive engagement strategies, involving patients more directly in requirements validation and prioritization through adapted participatory approaches.

The proposed methodology focused primarily on process-related outcomes, including stakeholder adoption and perceived usefulness, rather than direct measurement of clinical performance. At the same time, it supports verification and monitoring throughout the system lifecycle, enabling traceability between stakeholder needs, system requirements, and evaluation activities. This provides a structured basis for linking design decisions to downstream outcomes such as usability, adoption, and trust. However, establishing a causal link between requirements engineering decisions and clinical performance remains challenging, as outcomes are influenced by multiple subsequent design, data, and implementation factors. Therefore, the present study demonstrates the feasibility and practical value of the participatory requirements engineering approach rather than its isolated impact on clinical effectiveness. Future research should explore the longitudinal impact of structured, stakeholder-driven requirements elicitation on the safety, performance, and clinical adoption of AI health solutions, including how early-stage engagement influences outcomes, such as regulatory readiness, clinician trust, and patient satisfaction. Comparative analyses between projects using participatory requirement frameworks and those relying on traditional, top-down approaches would generate valuable insights into the measurable benefits of inclusivity and structure in AI system design. Additionally, investigating methods to quantify stakeholder influence through metrics such as traceability of requirements, iteration frequency, or convergence of priorities would help operationalize the concept of “participatory rigor” within AI development.

The requirements engineering process presented in this study follows a structured, early-stage design approach that may resemble traditional software engineering models. While suitable for eliciting and prioritizing stakeholder needs, such approaches do not by themselves address lifecycle challenges specific to AI systems, including data drift and concept drift arising from evolving clinical and operational environments. The proposed framework should therefore be understood as an initial phase within a broader iterative lifecycle, where requirements are periodically revisited and updated through postdeployment monitoring, stakeholder feedback, and model evaluation processes. Future work should explore mechanisms for integrating continuous feedback and adaptive requirement revision throughout the AI system lifecycle.

Another promising avenue lies in adapting the methods here proposed to emerging paradigms in health care AI, including federated learning, multimodal data integration, and adaptive clinical decision-support systems. Cross-sectoral studies would further test the process’s robustness in varied regulatory and cultural contexts, contributing to the development of international standards for ethical and transparent AI requirements engineering. Together, these lines of investigation would consolidate the scientific and practical foundations of participatory, structured approaches to AI innovation in health care and beyond.

In conclusion, the methodology developed and hereby presented, despite its limitations, extends traditional techniques for requirements elicitation by including SI and multistakeholder engagement and ways to systematically incorporate nontechnical requirements from diverse viewpoints, contexts, and disciplines. The framework aims to support the development of AI solutions that are not only technically robust but also clinically, legally, and socially trustworthy and relevant, serving as a practical guide for researchers and developers aiming to align AI innovation with real-world health care needs.

## Supplementary material

10.2196/87984 Multimedia Appendix 1 Radioval requirements catalog.

## Abbreviations

AI: artificial intelligence
CE: Conformité Européenne
ELSI: ethical, legal, and social implications
EU: European Union
FUTURE-AI: fairness, universality, traceability, usability, robustness, and explainability in AI
GA: Grant Agreement
IEC: International Electrotechnical Commission
IEEE: Institute of Electrical and Electronics Engineers
ISO: International Organization for Standardization
MDR: Medical Device Regulation
MoSCoW: Must have, Should have, Could have, Will not have
RadioVal: International Clinical Validation of Radiomics Artificial Intelligence for Breast Cancer Treatment Planning
SI: social innovation
